# Magnesium enhances the chondrogenic differentiation of mesenchymal stem cells by inhibiting activated macrophage-induced inflammation

**DOI:** 10.1038/s41598-018-21783-2

**Published:** 2018-02-21

**Authors:** Tu Hu, Haitao Xu, Chongyang Wang, Hui Qin, Zhiquan An

**Affiliations:** 1 Department of Orthopedics, Shanghai Jiao Tong University Affiliated Sixth People’s Hospital, Shanghai, China; 2 Trauma Center, Shanghai General Hospital, Shanghai Jiao Tong University School of Medicine, Shanghai, China

## Abstract

Magnesium deficiency increases the generation of pro-inflammatory cytokines, which is consistently accompanied by the sensitization of cells such as neutrophils, macrophages and endothelial cells. We investigated the potential of magnesium to regulate macrophage polarization and macrophage-induced inflammation with or without lipopolysaccharide (LPS) and interferon-γ (IFN-γ) activation and further elucidated whether these effects impact the inhibitory functions of activated macrophage-induced inflammation on cartilage regeneration. The results showed that magnesium inhibited the activation of macrophages, as indicated by a significant reduction in the percentage of CCR7-positive cells, while the percentage of CD206-positive cells decreased to a lesser degree. After activation, both pro-inflammatory and anti-inflammatory cytokines were down-regulated at the mRNA level and certain cytokines (IL-1β, IL-6 and IL-10) were decreased in the cell supernatant with the addition of magnesium. Moreover, magnesium decreased the nuclear translocation and phosphorylation of nuclear factor-κB (NF-κB) to impede its activation. A modified micromass culture system was applied to assess the effects of activated macrophage-conditioned medium with or without magnesium treatment on the chondrogenic differentiation of human bone marrow mesenchymal stem cells (hBMSCs). Magnesium enhanced the chondrogenic differentiation of hBMSCs by reversing the adverse effects of activated macrophage-induced inflammation.

## Introduction

Due to a limited ability to achieve self-repair and regeneration as well as a lack of effective therapeutic options, the degeneration and injury of articular cartilage may result in severe consequences^1^. The emergence of autologous chondrocyte implantation has challenged the theory of William Hunter, who proposed that damaged cartilage cannot be reconstituted, which has been regarded as an axiom for approximately 250 years^2,3^. However, apart from the major limitation that newly synthesized cartilage is formed by fibrous rather than hyaline chondrocytes, whose biomechanical and physiological capacities are not comparable to those of initial articular cartilage, other problems, such as additional damage to the non-weight-bearing region and insufficient cell expansion, also restrict the application of this method. Recently, stem cells have aroused interest as a promising alternative cell source for treating articular cartilage defects, as these cells differentiate into chondrocytes under conditioned circumstances^4^. The more extensive availability of mesenchymal stem cells (MSCs) from various tissues, such as bone marrow^5^, adipose tissue^6^, synovial membrane^7^ and other tissues, compared with that of original chondrocytes, as well as the higher proliferative capacity of MSCs, highlight the attractiveness of MSCs as cell substitutes in cartilage regeneration.

Macrophages are important components of innate immunity and play an essential role in inflammation and accompanying host defence^8^. Macrophages have the potential to switch phenotypes according to different environmental cues, exhibiting a high degree of plasticity. The classically activated M1 phenotype, with typical surface markers CD11c and C-C chemokine receptor type 7 (CCR7, also known as CD197), enhances T helper cell 1 (Th1) type inflammation^9^. The alternatively activated M2 phenotype, with the typical surface markers CD163 and CD206, enhances T helper cell 2 (Th2) type inflammation, down-regulates inflammation and improves tissue healing^8^. Many studies have shown that macrophages with anti-inflammatory and tissue repair phenotypes exert a chondro-inductive effect when co-cultured with MSCs, while macrophages with a pro-inflammatory phenotype do not transmit chondro-inductive signals or even inhibit the chondrogenic differentiation of MSCs^10–13^.

Magnesium (Mg) performs a variety of biological functions as the second-most abundant cation in cellular systems. Moreover, magnesium alloy has been recognized as a promising metal for orthopaedic implant applications based on its induction of osteochondral regeneration; furthermore, the local application of magnesium might dissuade concerns about hypermagnesaemia^14,15^. Previous studies have shown that magnesium deficiency results in cartilage lesions^16^ and increases the production of interleukin (IL)−6^17^, a pro-inflammatory cytokine that plays a critical role in osteoarthritis (OA)^18^. Additionally, magnesium strengthens the adherence and cartilage differentiation of synovial MSCs through integrins in a dose-dependent manner^19^. Sugimoto *et al*. found that magnesium increased basal IκB-α (nuclear factor of kappa light polypeptide gene enhancer in B-cells inhibitor, alpha) levels and reduced the production of inflammatory cytokines, such as tumour necrosis factor-α (TNF-α) and IL-6, in intrapartum women as well as term and preterm neonates, indicating that magnesium exerts a significant impact on human immunoregulation^20^. However, whether the immunoregulatory effects of magnesium on macrophages affect inflammation-induced chondrocyte regeneration has not been fully investigated to date.

According to previous studies^10,13^ indicating that magnesium plays a positive role in the chondrogenic differentiation of MSCs, whereas macrophages with a pro-inflammatory phenotype have the opposite effect, we wondered whether magnesium affects the inflammation induced by macrophages and, furthermore, whether this effect influences the chondrogenic differentiation of MSCs. In the present study, we first examined the potential effects of magnesium on the phenotypic changes in macrophages and their release of inflammatory cytokines with or without lipopolysaccharide (LPS) and interferon-γ (IFN-γ) activation. Then, we investigated the effects of magnesium on the nuclear translocation of nuclear factor-κB (NF-κB) induced by LPS and IFN-γ in RAW 264.7 (RAW) cells to validate its anti-inflammatory mechanism. Finally, we investigated the chondrogenic differentiation of human bone marrow MSCs (hBMSCs) co-cultured with activated macrophage cell-conditioned medium and the potential effects of magnesium addition on this process. The results elucidate a novel immunoregulatory pathway for magnesium in chondrogenesis and provide foundational data for the application of magnesium-containing implants in cartilage regeneration to achieve better clinical outcomes.

## Results

### Impact of magnesium on cell viability

**Figure 1:**
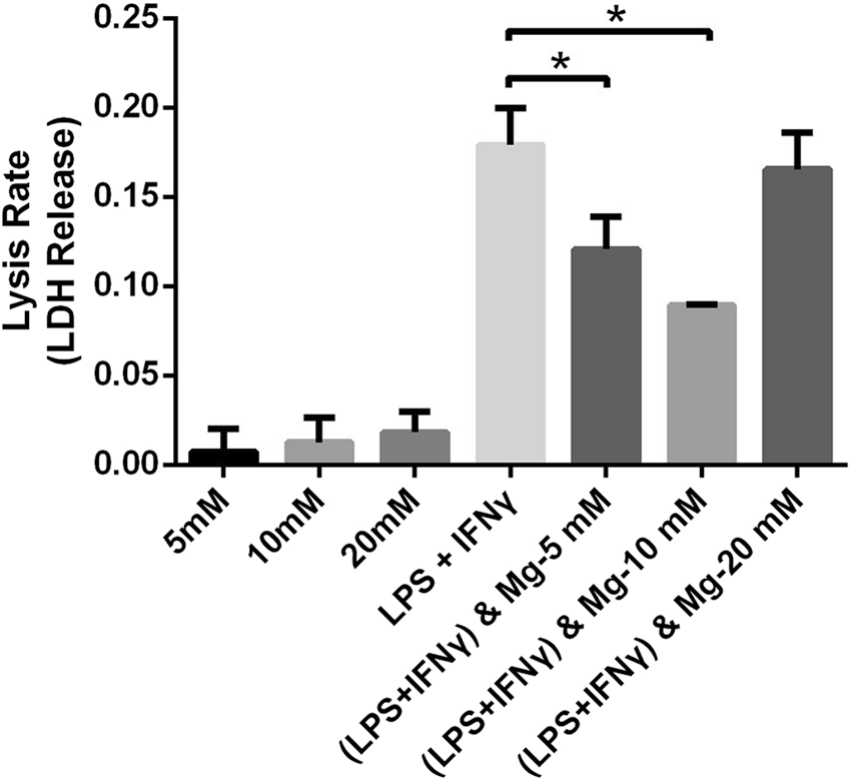
Lysis rates of RAW cells treated with magnesium in the absence or presence of LPS and IFN-γ for 3 days were measured by LDH release assay. (**p < 0.01; ***p < 0.001).

A lactate dehydrogenase (LDH) release assay was performed to investigate the cytotoxic effects of magnesium on RAW cells in the absence or presence of LPS and IFN-γ. With increasing magnesium concentrations, the lysis rate of RAW cells showed no obvious change and maintained nearly normal levels (Fig. 1). However, treatment with LPS and IFN-γ for 3 days significantly increased the lysis rate of RAW cells, suggesting that LPS and IFN-γ induce cell death and destruction. Interestingly, the increased lysis rate of RAW cells caused by LPS and IFN-γ obviously decreased after the addition of 5 and 10 mM magnesium, potentially implying that magnesium exerts cytoprotective effects against external injury.

### Effects of magnesium on the phenotype switch and cytokine production of RAW cells

**Figure 2:**
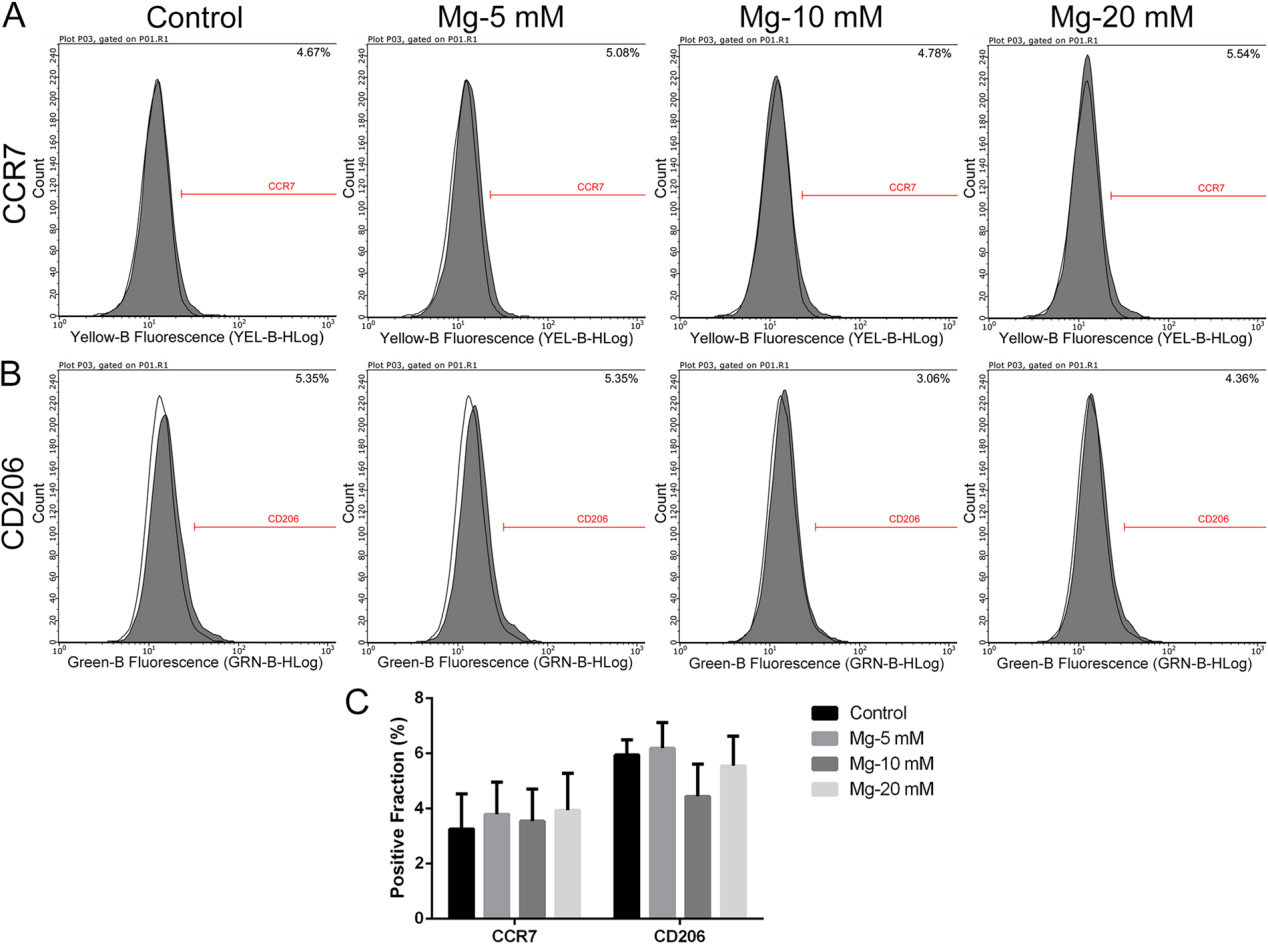
The effects of magnesium on the phenotype switch of non-activated macrophages. (**A** and **B**) Representative histograms of flow cytometric results: percentages of CCR7- or CD206-positive cells, representing M1 or M2 macrophages, respectively. The isotype controls are shown as none-filled histograms. (**C**) Statistical results for CCR7- or CD206-positive macrophages from three repeated experiments.

**Figure 3:**
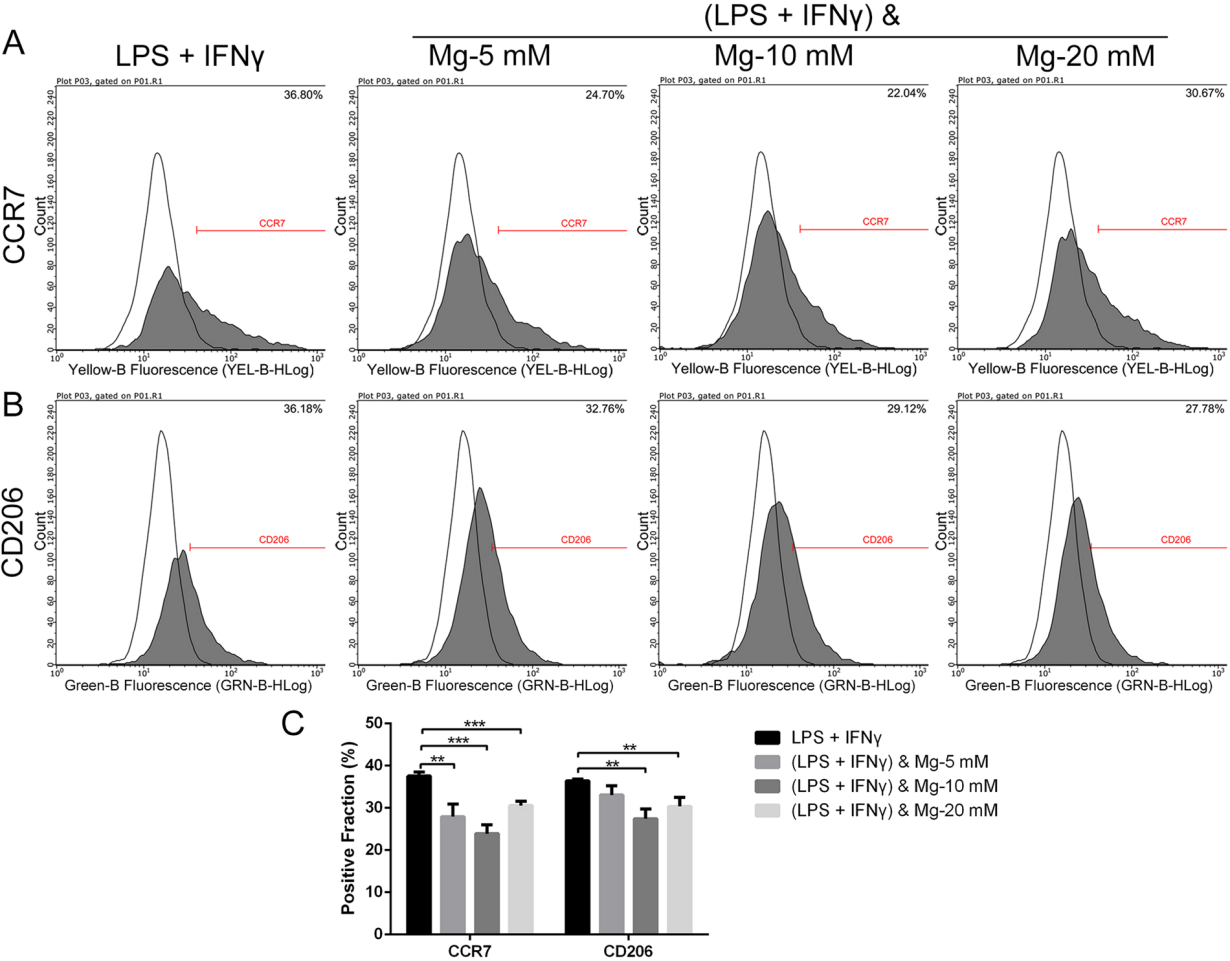
The effects of magnesium on the phenotype switch of macrophages upon activation with LPS and IFN-γ. (**A** and **B**) Representative histograms of flow cytometric results: percentages of CCR7- or CD206-positive cells, representing M1 or M2 macrophages, respectively. (**C**) Statistical results for CCR7- or CD206-positive macrophages from three repeated experiments. (**p < 0.01; ***p < 0.001).

Flow cytometry was used to evaluate the effects of magnesium on the phenotype switch of RAW cells. Representative histograms of CCR7 (an M1 marker) and CD206 (an M2 marker) under different treatment conditions are shown in Figs 2 and 3. The statistical results for the CCR7- and CD206-positive fractions from flow cytometry are shown in Figs 2C and 3C. Different concentrations of magnesium (5, 10 and 20 mM) did not notably affect the percentages of CCR7- and CD206-positive cells compared with those of the control group (Fig. 2). After stimulation with LPS and IFN-γ, the percentages of both CCR7- and CD206-positive cells increased (Fig. 3). Interestingly, 5 mM magnesium inhibited the effects of LPS and IFN-γ, with a significant reduction in the percentage of CCR7-positive cells, while the percentage of CD206-positive cells did not change substantially. Magnesium supplementation to 20 mM also decreased the expression of CCR7 in RAW cells, but the rate reduction was lower than that in the groups treated with 5 and 10 mM magnesium. Due to the damage caused by LPS and IFN-γ to cells, as shown in Fig. 1, the fluorescence intensities of these two markers in cells stimulated with LPS and IFN-γ dramatically decreased (Fig. 3A,B) compared with those in the groups treated with magnesium only. In addition, as shown in Supplementary Fig. S1, the expression of CD206 in RAW cells was increased by IL-4 stimulation. Magnesium addition at different concentrations only slightly decreased the percentages of CD206-positive cells compared with those of the cells stimulated only by IL-4, and no significant difference was found. Based on the findings described above, 5 mM magnesium was used for the following studies. Throughout the remainder of the results, the concentration of magnesium is 5 mM unless otherwise specified.

**Figure 4:**
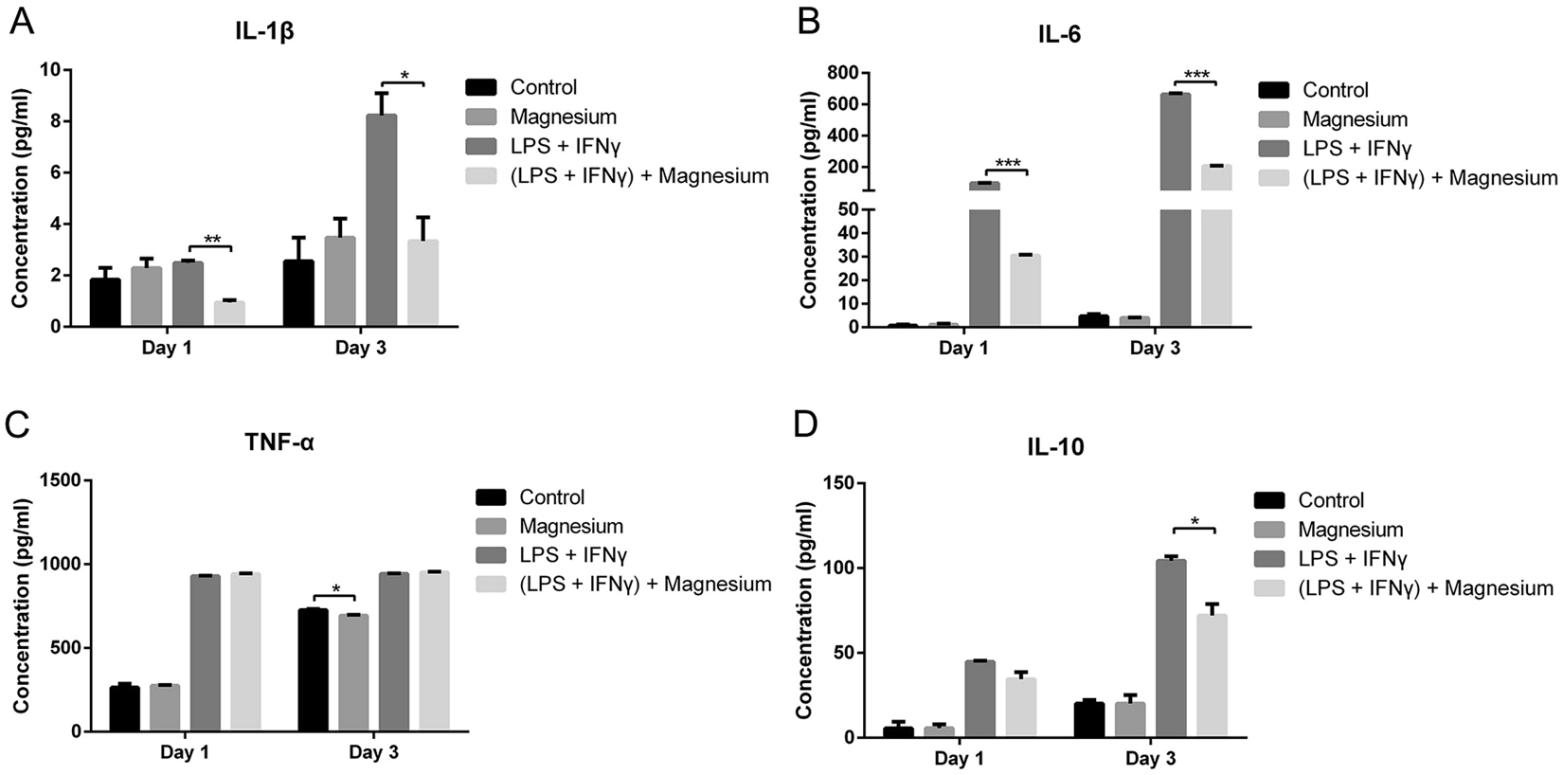
ELISA results for inflammatory cytokine production by RAW cells at days 1 and 3: (**A**) IL-1β; (**B**) IL-6; (**C**) TNF-α; (**D**) IL-10. (*p < 0.05; **p < 0.01; ***p < 0.001).

Cytokine levels determined by enzyme-linked immunosorbent assay (ELISA) are shown in Fig. 4. As we wanted to test the short and long-term effects of magnesium on macrophage-mediated inflammation upon LPS and IFN-γ stimulation, we measured cytokine levels on days 1 and 3. With the addition of LPS and IFN-γ, the concentrations of IL-1β, IL-6, TNF-α and IL-10 were all increased. At both days 1 and 3, magnesium restrained the production of IL-1β and IL-6 in the group stimulated by LPS and IFN-γ. However, in the groups without LPS and IFN-γ treatment, magnesium had no significant effect on the levels of these two cytokines (Fig. 4A,B). Unlike IL-1β and IL-6, TNF-α levels were not affected by magnesium when treated with LPS and IFN-γ (Fig. 4C). Similar to IL-1β and IL-6, magnesium did not alter the concentration of the anti-inflammatory cytokine IL-10 compared to that in the negative group; however, after boosting with LPS and IFN-γ, IL-10 levels significantly decreased by day 3 (Fig. 4D).

**Figure 5:**
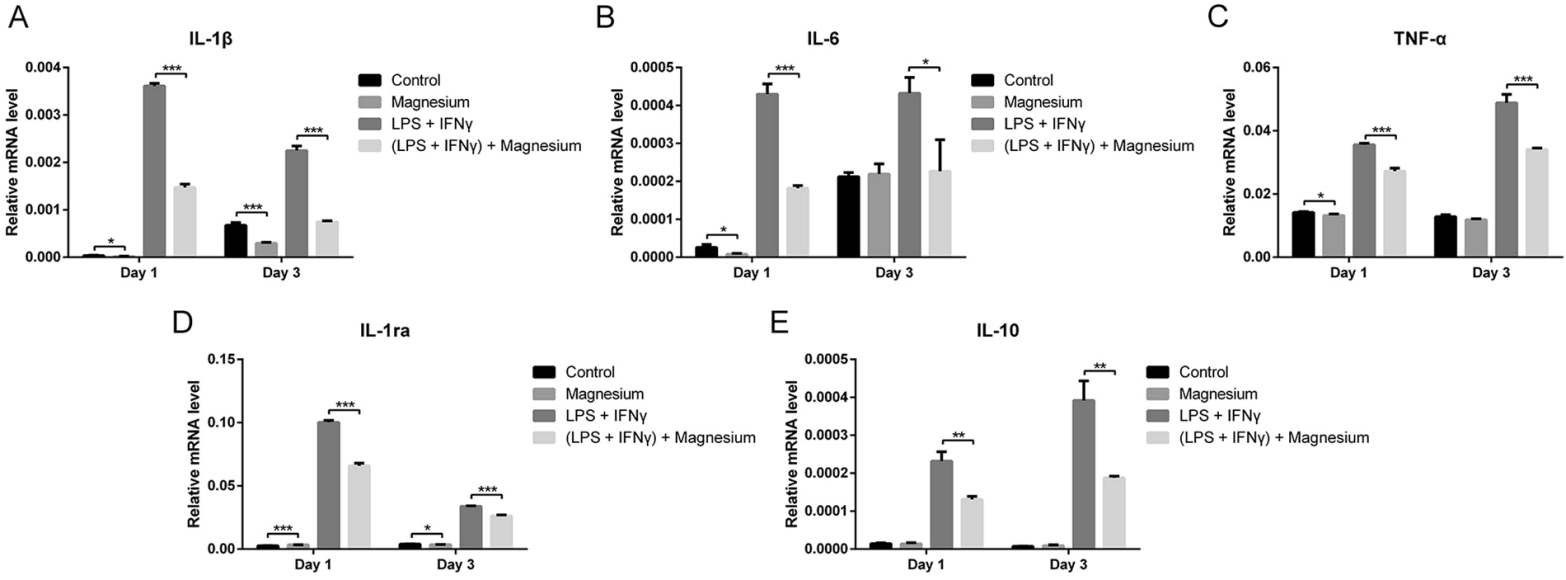
Relative mRNA expression levels of inflammatory cytokines in RAW cells at days 1 and 3: (**A**) IL-1β; (**B**) IL-6; (**C**) TNF-α; (**D**) IL-1ra; (**E**) IL-10.

With LPS and IFN-γ stimulation, the mRNA expression of the pro-inflammatory (TNF-α, IL-6 and IL-1β) and anti-inflammatory (IL-1ra and IL-10) genes was significantly up-regulated on both days 1 and 3 (Fig. 5). Upon magnesium treatment, the mRNA expression of IL-1β and IL-6 was down-regulated, and this trend became more obvious after simulation with LPS and IFN-γ (p < 0.05) (Fig. 5A,B). TNF-α showed the same trend as IL-1β and IL-6 (Fig. 5C), which differed from the results obtained by ELISA. No significant difference was found in the expression of IL-10 following treatment with magnesium only, whereas a notable reduction was observed when LPS and IFN-γ were involved (p < 0.05) (Fig. 5E).

### Magnesium decreases cytokine production by reducing NF-κB activation

**Figure 6:**
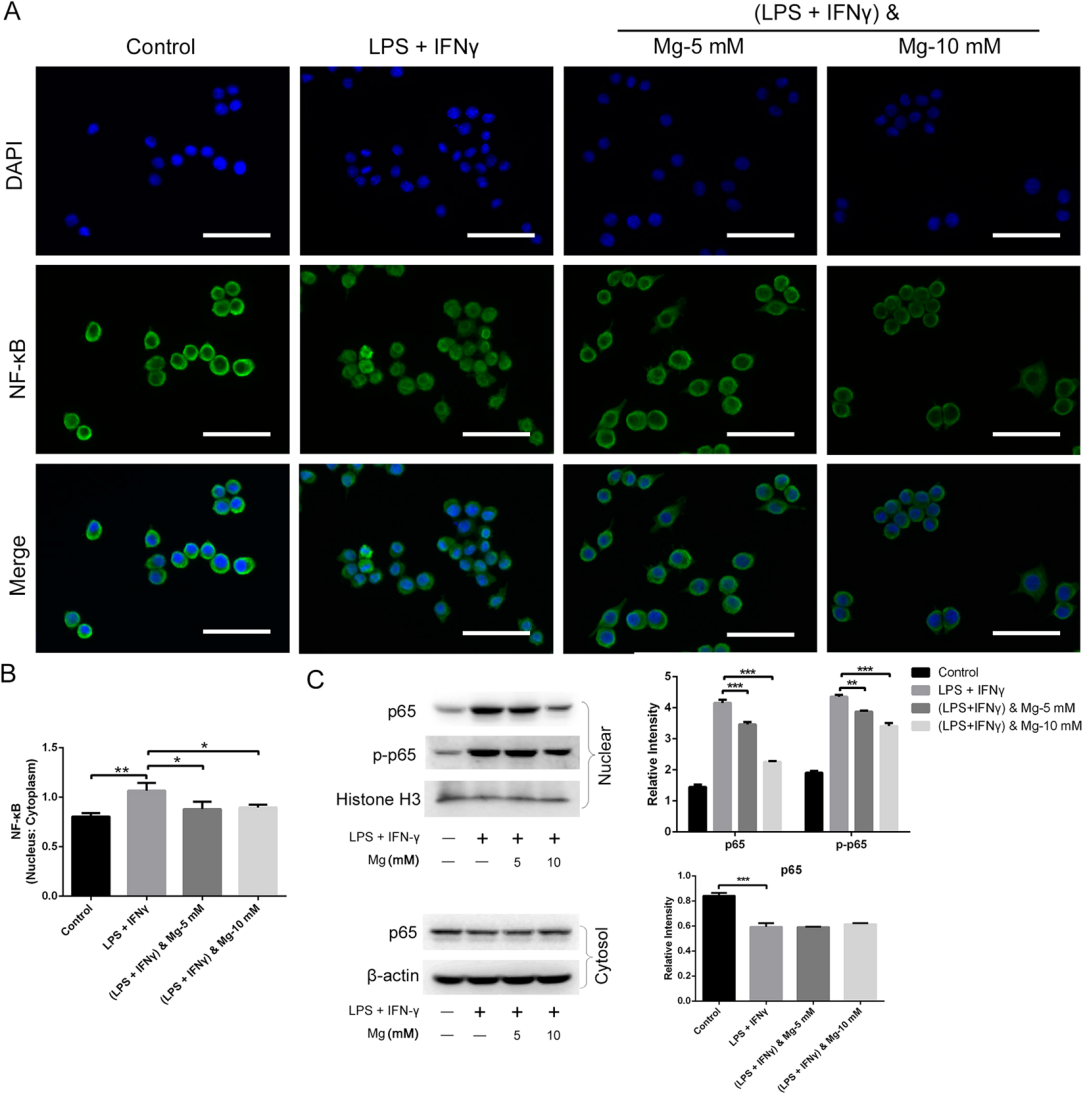
Effects of magnesium on nuclear NF-κB translocation in RAW cells activated by LPS and IFN-γ. (**A**) Nuclear translocation of NF-κB was observed by fluorescence microscopy. Scale bar = 50 μm. (**B**) The nuclear:cytoplasmic ratio of p65 was measured as a relatively quantitative evaluation of NF-κB nuclear translocation. (**C**) Immunoblotting for NF-κB p65 and phosphorylated NF-κB p65 (p-p65) was performed using the cytoplasmic and nuclear fractions. The bar chart shows the quantitative evaluation of protein bands by densitometry and the levels are presented as the mean ± SD. (*p < 0.05; **p < 0.01).

Upon activation through LPS and IFN-γ, the enzyme IκB kinase (IKK) phosphorylates the IκB-α protein, leading to its dissociation from NF-κB and eventual degradation by the proteosome. The activated NF-κB complex is free to enter the nucleus and then stimulate the expression of several inflammatory genes^21^. Fluorescence microscopy was used to observe the cellular distribution of NF-κB, revealing that LPS together with IFN-γ significantly promoted the nuclear translocation of NF-κB p65, in accordance with the findings of previous studies^21,22^. Non-activated RAW cells exhibited relatively low levels of NF-κB in the nucleus, as demonstrated by the low nuclear:cytoplasmic ratio (0.80). Increased NF-κB translocation to the nucleus was observed upon stimulation with LPS (10 ng/mL) and IFN-γ (10 ng/mL) for 30 min (1.07). However, with the prior addition of magnesium (5 and 10 mM) for 2 h, the elevated nuclear translocation of NF-κB was inhibited in LPS- and IFN-γ-stimulated cells, with decreases in the nuclear:cytoplasmic ratio to 0.88 and 0.90, respectively (Fig. 6A,B).

We then performed immunoblotting to investigate whether magnesium attenuates the LPS- and IFN-γ-induced activation of the NF-κB signalling pathway. Magnesium pre-treatment inhibited the nuclear accumulation and phosphorylation of the NF-κB p65 subunit in a dose-dependent manner (Fig. 6C). Cytoplasmic levels of p65 were decreased following treatment with LPS and IFN-γ, but this effect was slightly inhibited in cells treated with magnesium supplementation (Fig. 6C). Altogether, these results suggest that magnesium inhibits the phosphorylation of NF-κB p65 and limits the translocation of NF-κB into the nucleus, resulting in the inhibition of NF-κB-mediated inflammation. The original gel photography of various protein extracts is shown in supplementary information.

### Effects of activated RAW cell-conditioned medium with or without magnesium treatment on the chondrogenic differentiation of BMSCs

**Figure 7:**
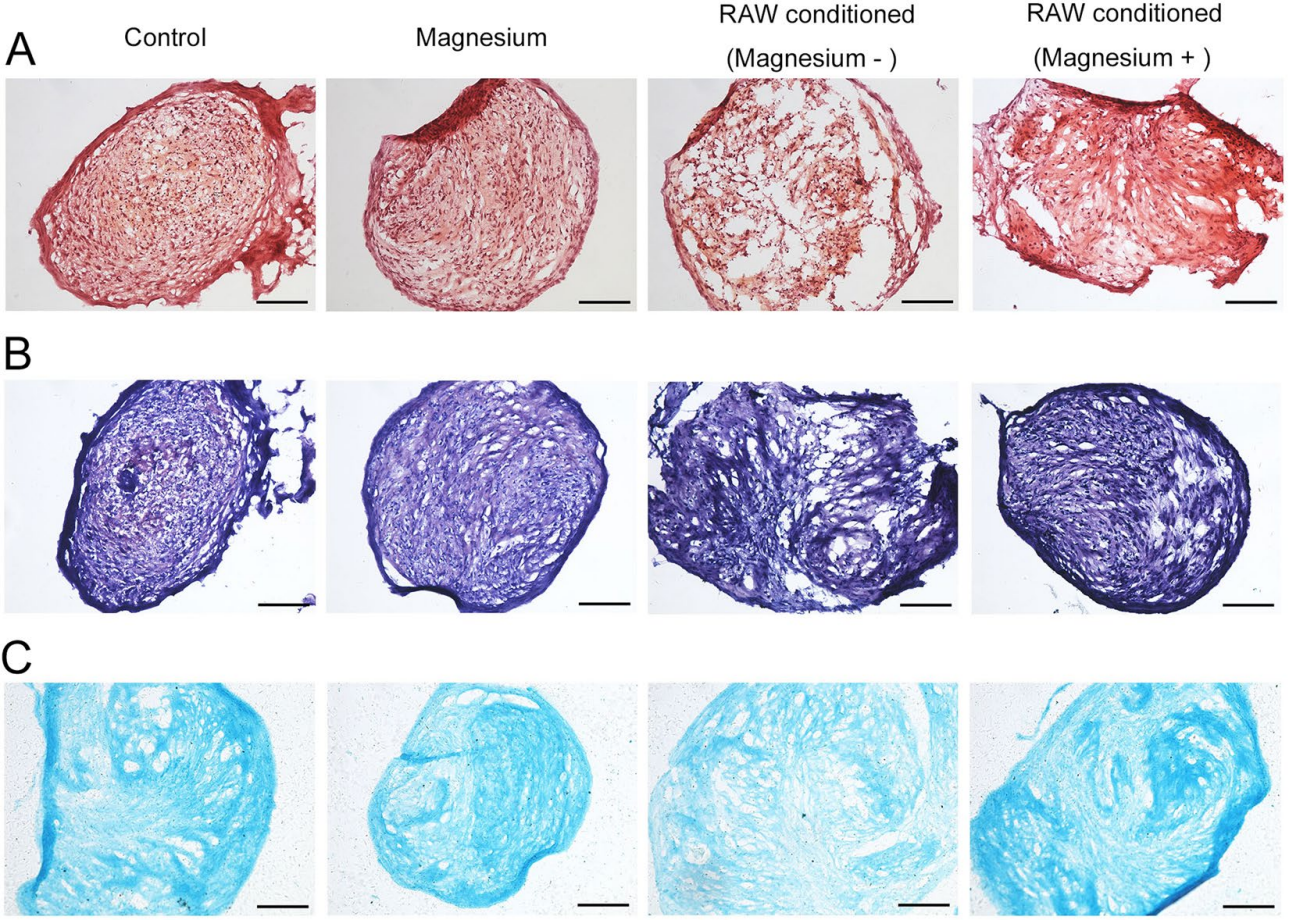
Histological staining of induced cartilage tissues from micromasses after culturing for 21 days. (**A**) Safranin O staining; (**B**) toluidine blue staining; (**C**) alcian blue staining. Scale bar = 100 μm.

**Figure 8:**
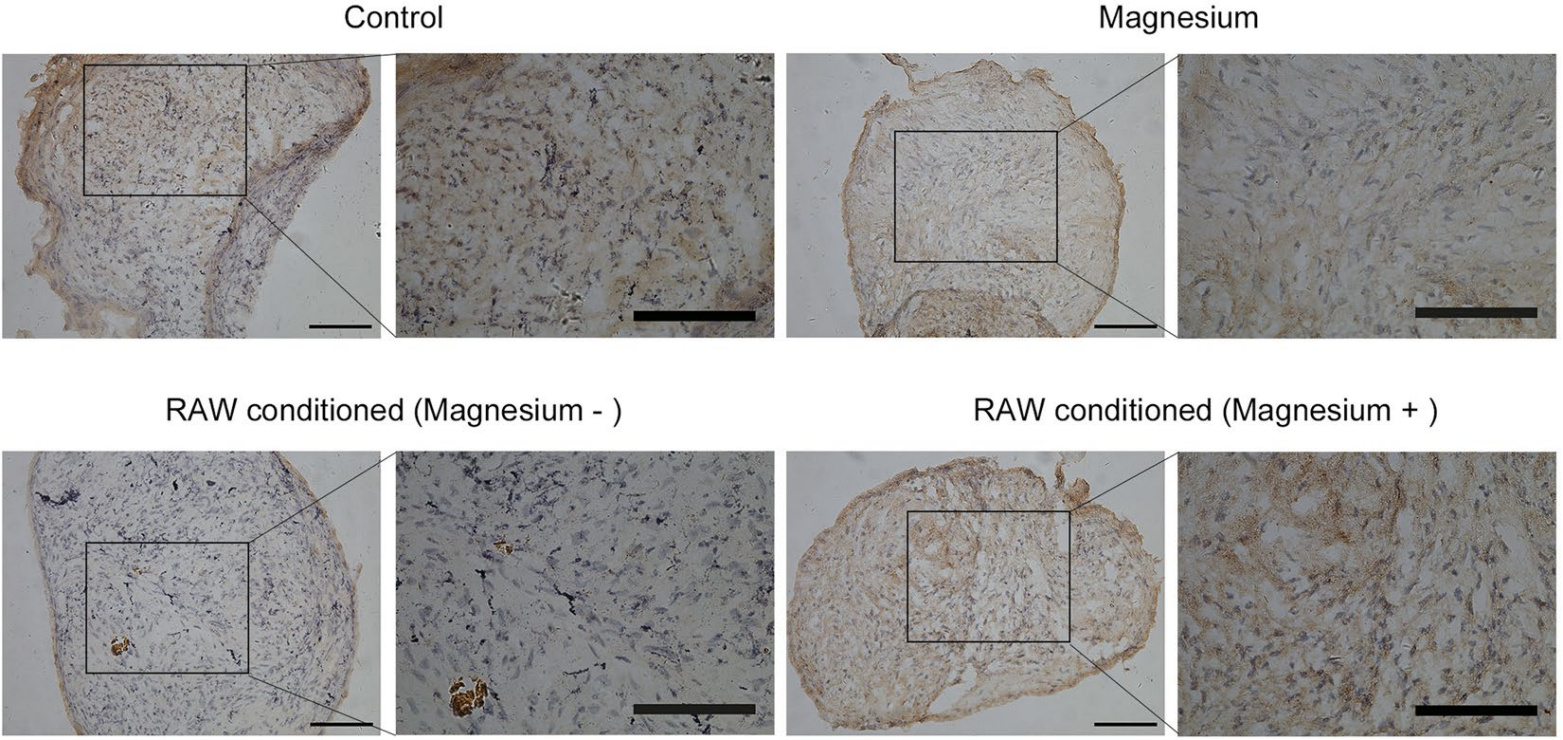
Immunohistochemical staining of induced cartilage tissues from micromasses for collagen type II after culturing for 21 days. Scale bar = 100 μm.

As the addition of magnesium (5 mM) to the culture medium of RAW cells increased the total magnesium concentration of the mixed culture medium of hBMSCs to approximately 2.2 mM, a treatment group with magnesium addition to 2.2 mM was used as another control. After three weeks of culturing, staining with safranin O (Fig. 7A), toluidine blue (Fig. 7B) and alcian blue (Fig. 7C) was performed to detect the production of glycosaminoglycans (GAGs), important components of proteoglycans in the extracellular matrix (ECM). The electrostatic binding of cationic dyes to the anionic groups (primarily sulphates) of GAGs in the sections^23^ confirmed that the hBMSCs were successfully induced to cartilaginous tissue. No obvious differences were found in staining intensity and tissue morphology between the magnesium-added group (2.2 mM) and the control group, suggesting that this concentration of magnesium exerted no clear effects on proteoglycan production. However, in the activated RAW cell-conditioned group, the staining was more sparse and disordered than that of the control group, indicating that less proteoglycan was formed. Intriguingly, the effects of the activated macrophage-conditioned medium on the chondrogenic differentiation of hBMSCs were reversed by magnesium treatment on macrophages. Immunohistochemical staining for collagen type II (Fig. 8) showed a trend similar to those observed in the three staining experiments mentioned above (images of the negative control are shown in Supplementary Fig. S2). The accumulation of collagen type II in the magnesium-added group showed no great difference compared with that in the control group, but lower accumulation was clearly evident in the activated RAW cell-conditioned group. Similarly, the accumulation of collagen type II in the activated RAW cell-conditioned group was reversed by the addition of magnesium.

**Figure 9:**
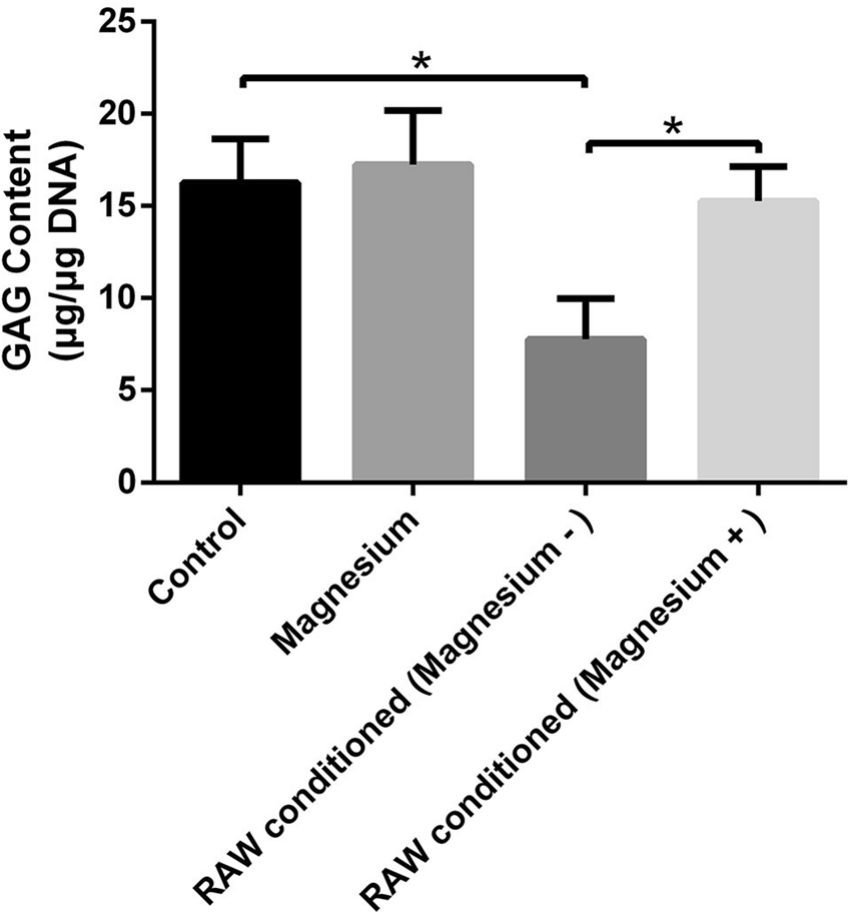
GAG content (μg) normalized against DNA content (μg). (*p < 0.05).

The sulphated glycosaminoglycan (sGAG) content was used as a measurement of proteoglycan produced by hBMSCs (Fig. 9). After three weeks of culturing, the sGAG content in the group treated with magnesium (2.2 mM) showed no significant difference from that in the control group. Although the activated RAW cell-conditioned group showed a significant decline in sGAG content compared with the control group, the addition of magnesium to RAW cell-conditioned medium had an obvious inhibitory effect on this process (p < 0.05).

**Figure 10:**
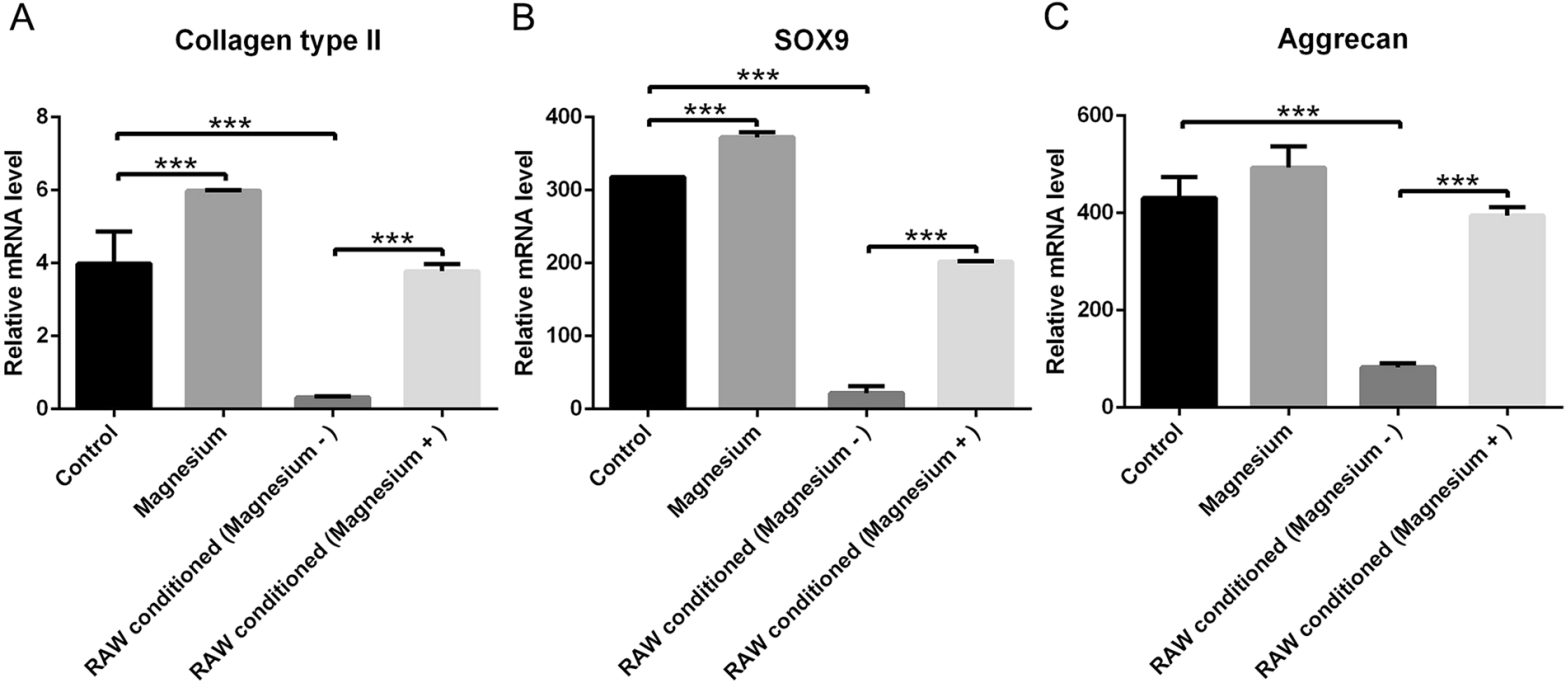
Relative mRNA expression levels of collagen type II (**A**), SOX9 (**B**) and aggrecan (**C**). (***p < 0.001).

Quantitative real-time polymerase chain reaction (RT-qPCR) was applied to determine chondrogenic gene expression (Fig. 10). After culturing for 21 days, magnesium (2.2 mM) slightly enhanced the expression of collagen type II and SOX9 (Fig. 10A,B), but no obvious effect on aggrecan expression was observed (Fig. 10C). The expression of SOX9, aggrecan and collagen type II in hBMSCs treated with the conditioned medium of activated RAW cells was statistically lower than that of the control group (Fig. 10A,B and C). Moreover, the addition of magnesium to the medium of activated RAW cells significantly reversed this process, increasing the expression of three chondrogenic genes in hBMSCs.

## Discussion

Several studies have shown that magnesium deficiency increases the production of many pro-inflammatory cytokines (such as TNF-α, IL-6) and that this effect is accompanied by the sensitization of cells such as neutrophils, macrophages and endothelial cells^14,17,24–26^. The development of cartilage injury-induced arthritis involves a series of immune responses that mobilize various types of immune cells into the wound site and activate host defences and tissue repair. Among these cells, macrophages have received considerable attention as modulators promoting better tissue remodelling^27^. The use of magnesium as an implant material represents a promising approach to cartilage regeneration due to its mechanical properties as well as its biodegradability^28^. Although many researchers have attempted to investigate the effects of biomaterials containing magnesium on changes in macrophage phenotypes and the secretion of inflammatory cytokines^14,27^, few studies have described the functions of pure magnesium ions. The present study investigated the effects of magnesium on macrophage polarization and the release of inflammatory cytokines from macrophages, with or without LPS and IFN-γ activation, and further elucidated whether these effects impact macrophage-induced cartilage regeneration.

RAW cells, in addition to being a mouse leukaemic cell line, possess some peculiarities but are a commonly used macrophage model demonstrating phenotype alterations (switching between M1 and M2) in response to environmental changes^9,29^. We thus selected RAW cells to facilitate the present investigation and found that magnesium at different concentrations had little effect on the phenotype changes in inactive macrophages. Whereas 5 and 10 mM magnesium significantly decreased the percentage of CCR7-positive cells in activated macrophages, 20 mM magnesium reduced CCR7 expression to a lesser degree, indicating that magnesium exerts a dose-dependent inhibition on M1 macrophage production. The expression of both CCR7 and CD206 increased, and the production of both pro- and anti-inflammatory cytokines was elevated in activated macrophages because LPS also activates CREB and C/EBPβ to induce the production of M2 macrophages in addition to promoting the generation of M1 macrophages through the NF-κB pathway^30^. This phenomenon is called endotoxin tolerance and may result in the dynamic transition of macrophages from a pro-inflammatory phenotype to an anti-inflammatory phenotype, thus reducing the risk of excessive inflammation induced by macrophages while maintaining fully active antimicrobial systems^30^. Alterations in cytokine levels, as measured by ELISA, further confirmed the effects of magnesium on the phenotype switches in macrophages. After stimulation with LPS and IFN-γ, IL-1β, IL-6 and IL-10 levels were notably increased and then inhibited by the addition of magnesium. Similar results were found at the mRNA level. After stimulation with LPS and IFN-γ, magnesium supplementation strongly decreased the mRNA expression of IL-1β and IL-6 (48–67%) at both days 1 and 3, while the decrease in TNF-α at the mRNA level was relatively mild (23–30%) at both time points. TNF-α concentrations, measured by ELISA, showed no difference between the LPS + IFN-γ group and the LPS + IFN-γ + magnesium group, likely due to the comparatively moderate changes in the mRNA expression levels between these two groups. Apart from the studies mentioned above, we also measured the mRNA levels of the anti-inflammatory cytokines IL-1ra and IL-10 and found that magnesium supplementation produced the same trend in these two anti-inflammatory cytokines as that observed for pro-inflammatory cytokines, potentially also resulting from endotoxin tolerance; these results are consistent with the ELISA and flow cytometry results.

Toll-like receptor 4 (TLR4), a member of the Toll family, plays an important role in the signal transduction of LPS in cells. Activated TLR4 induces the activation of NF-κB and mitogen-activated protein kinases (MAPKs) in RAW cells exposed to LPS^31,32^. NF-κB is an essential player in the transcriptional regulation of immune and inflammatory responses, resulting in the production of several inflammatory cytokines, including IL-1β, TNF-α and IL-6^22^. Sugimoto *et al*. found that the production of TNF-α and IL-6 in neonatal monocytes under constitutive and LPS-stimulated conditions was substantially reduced after pre-treatment with a clinically effective MgSO_4_ concentration for a short time, and this effect did not influence the viability or phagocytic function of monocytes^20^. LPS stimulation leads to a transient increase in the intracellular free Ca^2+^ concentration in macrophages due to intracellular Ca^2+^ release, which might be a result of extracellular Ca^2+^ influx^33^. The increased intracellular free Ca^2+^ concentration activates NF-κB in a PKC-dependent signalling pathway^33^. As a natural Ca antagonist, magnesium may antagonize Ca^2+^ entry by inhibiting Ca^2+^ influx through L-type voltage-dependent Ca channels to exert its effects on inflammation^34,35^. The present results were consistent with those of Jun *et al*. and other previous studies^27,36,37^. Exposure to magnesium was correlated with inhibited NF-κB p65 nuclear translocation and diminished phosphorylation levels following TLR stimulation, suggesting that magnesium may down-regulate TLR-induced inflammatory cytokine production in an NF-κB-dependent manner. Pro-inflammatory cytokines, such as TNF-α and IL-1β, may impede the chondrogenic differentiation of synovial fluid MSCs and aggravate cartilage degradation^38–41^. Although it appears attractive to use the *in situ* chondrogenesis of MSCs for cartilage repair, in many instances, this process requires an inflamed environment^40^. Many diseases are responsible for intra-articular inflammation, such as osteoarthritis and various traumas, including the iatrogenic trauma caused by cartilage repair surgery itself^40^. As TNF-α and IL-1β are dominant players in the development of local articular inflammation, the magnesium-mediated inhibition of macrophage-induced inflammatory cytokines might be a favourable factor for cartilage protection.

Extensive studies have shown that cartilage destruction induced by a magnesium-deficient diet in animals is identical to that induced by quinolones^42,43^. Magnesium directly enhances chondrocyte proliferation at a concentration of 10 mM and indirectly improves the chondrogenesis of human synovial MSCs by inhibiting the production of inflammatory cytokines and up-regulating the expression of cartilage formation-related genes^19,28,44^. Shimaya *et al*. showed that 5 mM magnesium exerted the greatest effects, enhancing the chondrogenesis of human synovial MSCs, but 2.5 mM showed no obvious effects^19^. Although expression of the chondrogenic-related genes collagen type II and SOX9 was slightly increased following treatment with 2.2 mM magnesium, the findings from the histological analysis are consistent with the results showing that a low concentration of magnesium (2.2 mM) had undetectable effects on chondrogenic induction, with no additional proteoglycan generation and no extra accumulation of collagen type II, which was validated by GAG quantification. Fahy *et al*. found that MSC chondrogenesis was inhibited by osteoarthritic synovium-conditioned medium through cytokines secreted by synovial macrophages, and M1-polarized subsets were considered potential mediators of these anti-chondrogenic effects^10^. Our results further validated the conclusion of Fahy, who showed that activated macrophages inhibited MSC chondrogenesis, thus resulting in lower proteoglycan generation, lower collagen type II accumulation, lower GAG production and lower expression of chondrogenic-related genes. Furthermore, magnesium involvement hindered the production of activated macrophages and their release of pro-inflammatory cytokines, confirming that magnesium inhibited activated macrophage-induced inflammation to provide a favourable environment for chondrogenesis. The results of chondrogenic-related staining showed that although there was no directly detectable enhancement of chondrogenesis, a low concentration of magnesium indirectly increased proteoglycan generation by inhibiting the adverse effects of activated macrophages. Similar results were obtained with the immunohistochemical analysis of collagen type II. Consistent with the qualitative results, GAG quantification and mRNA levels of collagen type II, SOX9 and aggrecan were all significantly increased in the group treated with activated RAW cell-conditioned medium with magnesium supplementation. There are certain potential shortcomings and limitations of the present study; for example, the precise molecular mechanism whereby magnesium reverses the adverse effects of macrophage-induced inflammation on chondrogenesis must be further investigated, and *in vivo* studies are also needed to confirm the findings described above.

In conclusion, the present results show that magnesium inhibition exerted effects on activated macrophages induced by LPS and IFN-γ and the secretion of inflammatory cytokines by these macrophages, potentially contributing to the anti-inflammatory properties of magnesium. Furthermore, magnesium enhanced the chondrogenic differentiation of MSCs by inhibiting the adverse effects of activated macrophage-induced inflammation.

## Materials and Methods

### Cell culture

The murine-derived macrophage cell line RAW 264.7 and hBMSCs were obtained from the Cell Bank of the Chinese Academy of Science. The RAW cells were maintained in Dulbecco’s modified Eagle’s medium (DMEM, Gibco, Grand Island, NY, USA), and the hBMSCs were maintained in α-minimum essential medium (αMEM, Gibco) supplemented with 10% foetal bovine serum (FBS, Gibco) and 1% penicillin/streptomycin (Gibco) at 37 °C in a humidified atmosphere of 5% CO_2_. The culture medium was changed every 3 days. The growing cells were passaged at approximately 80% confluence and only early passages (p3–5) for both cells were used in the present study.

### LDH release assay

The cytotoxic effects of magnesium (MgSO_4_) on RAW cells were measured using an LDH release assay as previously described^45^. Briefly, RAW cells were seeded onto 6-well plates (1 × 10^5^ cells per well) and treated with different concentrations of magnesium in the absence or presence of LPS (10 ng/mL, Sigma-Aldrich, St. Louis, MO, USA) and IFN-γ (10 ng/mL, PeproTech, Rocky Hill, NJ, USA) for 3 days. The high control group was treated with cell lysis buffer at 1 h prior to detection. After centrifugation, the supernatant (120 µL) from each well was transferred to a new 96-well plate and incubated with 60 µL of reaction mixture for 30 min in the dark according to the manufacturer’s instructions (Yeasen, Shanghai, China). Then, the absorbance was measured by a microplate reader at 490 nm. The cell lysis rate was calculated as follows: [(experimental − negative control)/(high control − negative control)] × 100%. MgSO_4_ (Amresco, Washington D.C., USA) was used as a source of magnesium ions for all experimental sections.

### Flow cytometry

Flow cytometry was used to detect the expression of the macrophage surface markers CCR7 (representing the M1 phenotype) and CD206 (representing the M2 phenotype). RAW cells were seeded at a density of 1 × 10^5^ cells per well (6-well plate) and treated with different concentrations of magnesium in the absence or presence of LPS (10 ng/mL) and IFN-γ (10 ng/mL). After stimulation for 24 h, the media from cells stimulated by LPS and IFN-γ with or without magnesium (5 mM) treatment were collected and centrifuged at 1500 rpm, and the obtained supernatants were stored at −80 °C for use in subsequent experiments with conditioned media. Cells from the control group, magnesium-addition groups (5, 10 and 20 mM) and groups treated with LPS + IFN-γ combined with different concentrations of magnesium were scraped from the wells, centrifuged and then resuspended in 1% bovine serum albumin (BSA) at room temperature for 30 min to block non-specific antigens. The samples were then incubated with phycoerythrin (PE)-conjugated anti-mouse CCR7 (eBioscience, San Diego, CA, USA) and fluorescein isothiocyanate (FITC)-conjugated anti-mouse CD206 (BioLegend, San Diego, CA, USA) for 1 h in the dark at ambient temperature in a final volume of 100 μL. After washing twice in 1% BSA, the cells were resuspended and analysed with a Guava flow cytometer (Millipore, Billerica, MA, USA). 5,000 cells were analysed in each test. FITC-conjugated rat IgG2a, κ and PE-conjugated rat IgG2a, κ were used as isotype controls. In addition, the expression of CD206 in cells stimulated by IL-4 (20 ng/mL, PeproTech) for 24 h with the addition of different concentrations of magnesium was also detected, and the results are shown in the supplementary data.

### ELISA

RAW cells were seeded at a density of 1 × 10^5^ cells per well (6-well plate). LPS (10 ng/mL), IFN-γ (10 ng/mL) and magnesium (5 mM) were added in the medium separately or together as planned. After 1 and 3 days, the medium was collected and centrifuged at 210 g to obtain supernatants for further ELISAs. The concentrations of IL-1β, IL-6, TNF-α and IL-10 were detected with ELISA kits (Anogen, Mississauga Ontario, Canada) according to the manufacturer’s instructions. The concentrations of these cytokines were calculated according to standard curves, and the obtained results are shown as the amount (pg) of IL-1β, IL-6, TNF- α and IL-10 per mL of supernatant.

### Quantitative real-time polymerase chain reaction

Total RNA from cells under different conditions was extracted using TRIzol reagent (Invitrogen, Carlsbad, CA, USA) according to the manufacturer’s protocol and quantified with a Nanodrop 2000 (Thermo Fisher Scientific, Waltham, MA, USA). First-strand cDNA was synthesized with a PrimeScript RT Reagent Kit (TaKaRa, Tokyo, Japan) and a MyCycler PCR (Bio-Rad, Hercules, CA, USA). RT-qPCR analysis was performed using an ABI PRISM®7900HT System. The following cycling conditions were used: 95 °C for 10 s, followed by 40 cycles of 95 °C for 5 s and 60 °C for 31 s using SYBR Green Premix Ex Taq (TaKaRa) with relative quantification methods. The relative expression levels of target genes were compared to those of β-actin (mouse) and GAPDH (human). The details of the primers used for RT-qPCR are summarized in Table 1.

**Table 1:** Primer pairs used in the RT-qPCR.

Genes	Primer sequences (5′ to 3′)
Human	
Sox 9	Forward: CTCTGGAGACTTCTGAACGAGAGC
	Reverse: GTTCTTCACCGACTTCCTCCG
Collagen type II	Forward: TGAAGGTGCTCAAGGTCCTC
	Reverse: ATTCCATCTGTTCCAGGGTT
Aggrecan	Forward: GCCAGCACCACCAATGTAAG
	Reverse: TTCAGTAACACCCTCCACGA
GAPDH	Forward: GGTGACTAACCCTGCGCTC
	Reverse: CAAATGAGCCCCAGCCTTCTC
Mouse	
IL-10	Forward: GAGAAGCATGGCCCAGAAATC
	Reverse: GAGAAATCGATGACAGCGCC
IL-1ra-	Forward: CTCCAGCTGGAGGAAGTTAAC
	Reverse: CTGACTCAAAGCTGGTGGTG
TNF-α	Forward: CTGAACTTCGGGGTGATCGG
	Reverse: GGCTTGTCACTCGAATTTTGAGA
IL-1β	Forward: TGGAGAGTGTGGATCCCAAG
	Reverse: GGTGCTGATGTACCAGTTGG
IL-6	Forward: ATAGTCCTTCCTACCCCAATTTCC
	Reverse: GATGAATTGGATGGTCTTGGTCC
β-actin	Forward: AGTGTGACGTTGACATCCGTA
	Reverse: GCCAGAGCAGTAATCTCCTTCT

### Immunofluorescence staining

The nuclear translocation of NF-κB p65 was detected by an immunofluorescence assay. For the present study, RAW 246.7 cells were pre-treated with magnesium (5 and 10 mM) for 2 h and then simultaneously stimulated with LPS and IFN-γ for 30 min. RAW cells were fixed with 4% paraformaldehyde diluted in PBS for 20 min, permeabilized with 0.5% Triton X-100 in PBS for 15 min, and blocked with 5% BSA for 1 h. The cells were probed with an anti-p65 NF-κB antibody (1:100, Cell Signaling Technology, Danvers, MA, USA) overnight at 4 °C and then incubated with FITC-conjugated goat anti-rabbit IgG (1:1000, Abcam, Cambridge, MA, USA) for 1 h at room temperature. The position of the cell nucleus was stained with 4,6-diamidino-2-phenylindole (DAPI, Sigma-Aldrich) solution (1 mg/mL) for 15 min. After washing with PBS, fluorescence was visualized using a fluorescence microscope (Leica Microsystems, Buffalo Grove, IL, USA). According to previous studies^46,47^, the intensity of p65 staining in the nucleus and cytoplasm was measured to determine the nuclear:cytoplasmic ratio as a relatively quantitative measurement of NF-κB nuclear localization.

### Western blot analysis

RAW 246.7 cells were pre-treated with magnesium (5 and 10 mM) for 2 h and then stimulated with LPS and IFN-γ for 30 min. The methods for protein extraction, followed by western blot analysis, were based on protocols from previous studies^22,48^. After washing with ice-cold PBS, the cytoplasmic and nuclear proteins of each sample were separately harvested using an extraction kit (Beyotime, Shanghai, China) to analyse the translocation of NF-κB into the nucleus. The protein extracts were centrifuged at 12,000 g for 15 min, and the protein contents of the supernatants were detected by performing a BCA protein assay (Beyotime). Approximately 20 μg of protein from each sample was loaded and separated on 10% SDS-polyacrylamide gels and then transferred onto polyvinylidene difluoride (PVDF) membranes (Millipore, Billerica, MA, USA). The membranes were incubated with 5% skim milk for 1 h at room temperature to block non-specific binding and then incubated with primary antibodies at 4 °C overnight. The next day, after washing with Tris-buffered saline containing 0.05% Tween 20 (0.05% TBST), the membranes were incubated with HRP-conjugated secondary antibodies (1:5000 dilution) for approximately 1 h. The signals were detected with an enhanced chemiluminescence (ECL) system after thorough washing with TBST. Histone H3 was used as internal control for the nuclear extracts, and β-actin was used as internal control for the cytoplasmic extracts. The primary antibodies used in the present study include anti-NF-κB p65 (dilution 1:1000, Cell Signaling Technology) and anti-phosphorylated NF-κB p65 (p-p65, dilution 1:1000, Cell Signaling Technology). ImageJ software was used to analyse the intensity of the bands in the western blot analysis.

### 3D-droplet pellet culture

A modified micromass culture system was adopted as previously described^49–51^. Briefly, the cells were harvested and resuspended in chondrogenic medium containing 10 ng/mL TGF-β1 (R&D Systems, Minneapolis, MN, USA), 100 nmol/L dexamethasone, 50 μg/mL ascorbate-2-phosphate, 40 μg/mL proline, 100 μg/mL pyruvate (all from Sigma), and 1:100 diluted ITS Premix (BD, East Rutherford, NJ, USA) at 1 × 10^7^ cells/mL. Cell droplets of 12.5 μL were carefully placed in a 24-well plate and incubated at 37 °C for 4 h to enable cell adherence, followed by the addition of 400 μL of chondrogenic medium and supernatants obtained from RAW cells or DMEM at a ratio of 2:1. After incubation for 24 h, the cell droplets aggregated and became spherical. The culture medium was changed every 3 days and micromasses were harvested on the 21st day.

### Histological analyses

After culturing for 3 weeks, the micromasses were fixed in 4% paraformaldehyde, followed by dehydration and embedding in Tissue-Tek® O.C.T. Compound (SAKURA, Torrance, CA, USA). Cross sections of 8 μm were obtained with a freezing microtome. The sections were individually stained with 0.5% safranin O (Sigma) solution, 0.5% toluidine blue (Sigma) solution and 1% alcian blue (Sigma) solution, and images were obtained with a light microscope (LEICA DM 4000 B, Leica Microsystems). These three cationic dyes were used to estimate the amounts of GAG, which was mostly produced by hyaline cartilage, in sections of induced cartilage.

For immunohistochemistry, the streptavidin–peroxidase-conjugation method was used as previously described^49^. After treatment with pepsin for 10 min at 37 °C, the sections were incubated with a peroxidase-blocking solution for 10 min and then reacted with mouse anti-human collagen type II monoclonal antibody (1:200, Invitrogen) at 4 °C overnight. After washing 3 times with PBS, the sections were incubated with biotin-conjugated secondary antibodies at room temperature for 10 min, followed by incubation for 10 min with streptavidin-conjugated peroxidase working solution. Subsequently, the sections were stained with 3, 3′-diaminobenzidine tetrahydrochloride (DAB) for 10 min, counterstained with Mayer’s haematoxylin for 1 min, dehydrated and then mounted for light microscopy evaluation. For negative controls, the primary antibody was substituted with PBS.

### GAG quantification

After culturing for 3 weeks, GAG content was assayed to quantitatively evaluate the effects of magnesium and activated RAW cell-conditioned medium with or without magnesium treatment on chondrogenesis according to a previously published method^52^. The micromasses were washed with PBS and digested by papain solution (1 mg/mL papain, 10 mM Na_2_EDTA and 10 mM L-cysteine hydrochloride dissolved in 0.1 M PBS) for 16 h at 57 °C. After digestion, the micromasses were centrifuged at 12,000 g for 3 min. The GAG content of each sample (n = 3) was quantitated by using a 1, 9-dimethylmethylene blue (DMMB, Sigma) dye binding assay, using a standard curve generated by chondroitin sulphate B. The absorbance was read at a wavelength of 525 nm. The DNA content in each sample lysate was determined by a PicoGreen® dsDNA quantitation assay. The GAG content was then normalized to the DNA content. The assay was performed in triplicate.

### Statistical analysis

All the statistical analyses were performed using Statistical Package for Social Sciences software (version 19.0) (SPSS, Inc., Chicago, IL, USA). All the results are shown as the mean ± standard deviation (SD). Data were analysed using one-way analysis of variance (ANOVA) with S-N-K post hoc t-tests. A value of 0.05 was set as the significance level, and the data were indicated with (∗) for p < 0.05.

### Data availability

The datasets generated and analysed during the current study are available from the corresponding author on reasonable request.

## Electronic supplementary material

Supplementary information
