# Supplementary material for: Magnesium enhances the chondrogenic differentiation of mesenchymal stem cells by inhibiting activated macrophage-induced inflammation
Source: Sci Rep. 2018 Feb 21;8:3406. doi: 10.1038/s41598-018-21783-2 (PMC5821731; doi:10.1038/s41598-018-21783-2)

Journal: Scientific Reports
Article ID: SREP-17-46405A

Title: Magnesium enhances the chondrogenic differentiation of mesenchymal stem cells by inhibiting activated macrophage-induced inflammation

Tu Hu†[1], Haitao Xu†[2], Chongyang Wang[1], Hui Qin[1], Zhiquan An*[1]

Supplementary Figure S1:

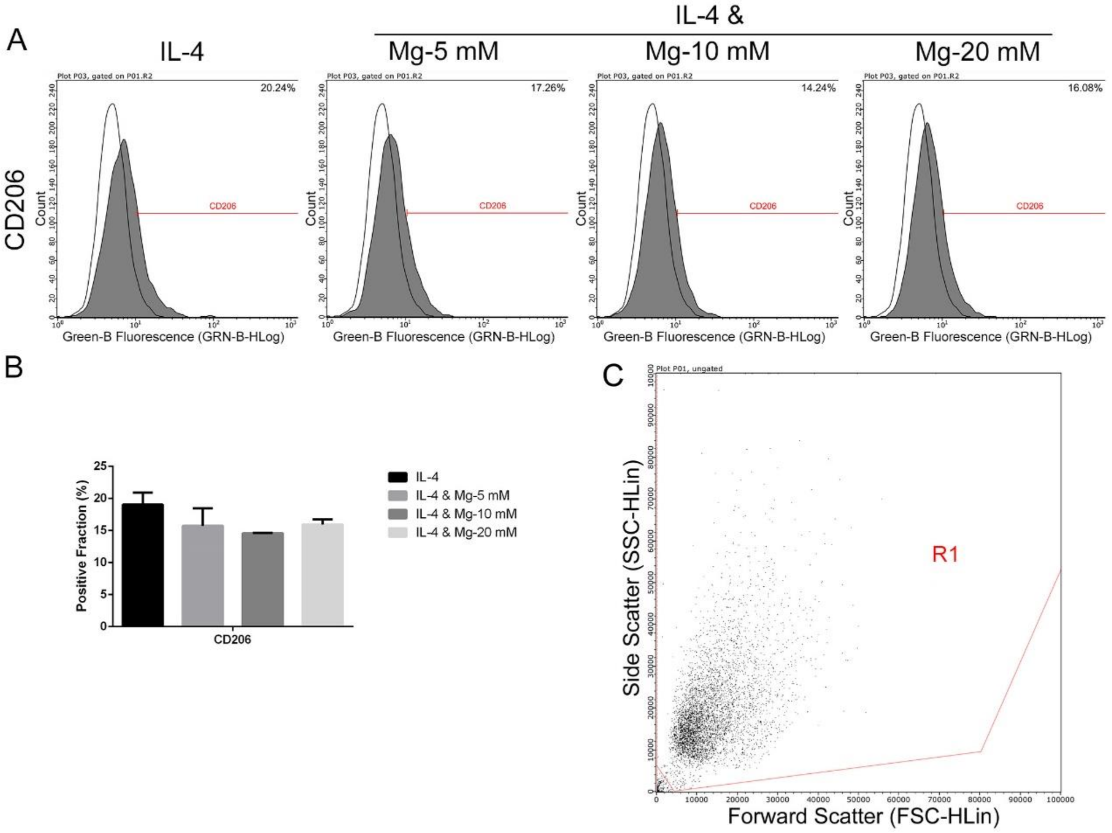

Supplementary Figure S1: The effects of magnesium on the phenotype change in macrophages upon stimulation with IL-4. **(A)** Histograms of the flow cytometric results, representing the percentage of CD206-positive cells. **(B)** Statistical results for CD206-positive macrophages from three repeated experiments. **(C)** A representative dot plot depicting the forward scatter (FSC) and side scatter (SSC) of RAW cells.

Supplementary Figure S2:

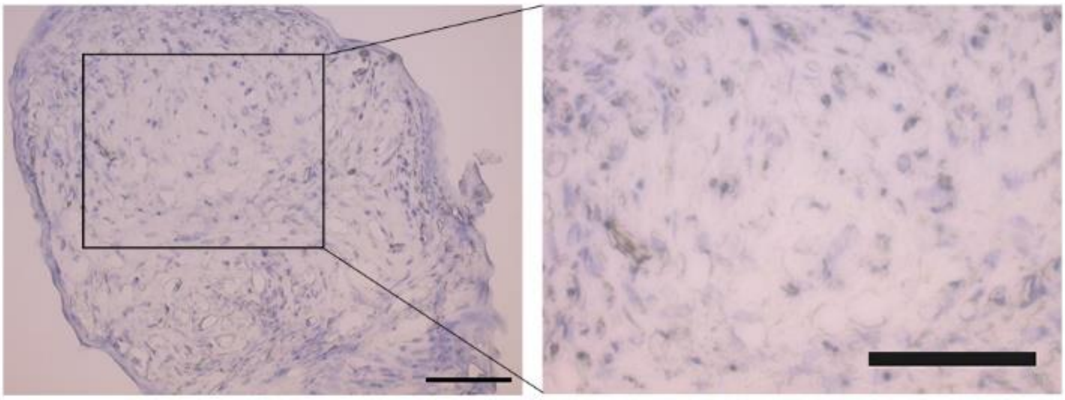

Supplementary Figure S2: Representative images of negative controls for the immunohistochemical staining of induced cartilage tissues from micromasses for collagen type II after culturing for 21 days. Scale bar = 100 μm.

Supplementary figures of western blot analysis

Gel photography of p65 protein in nuclear extracts:

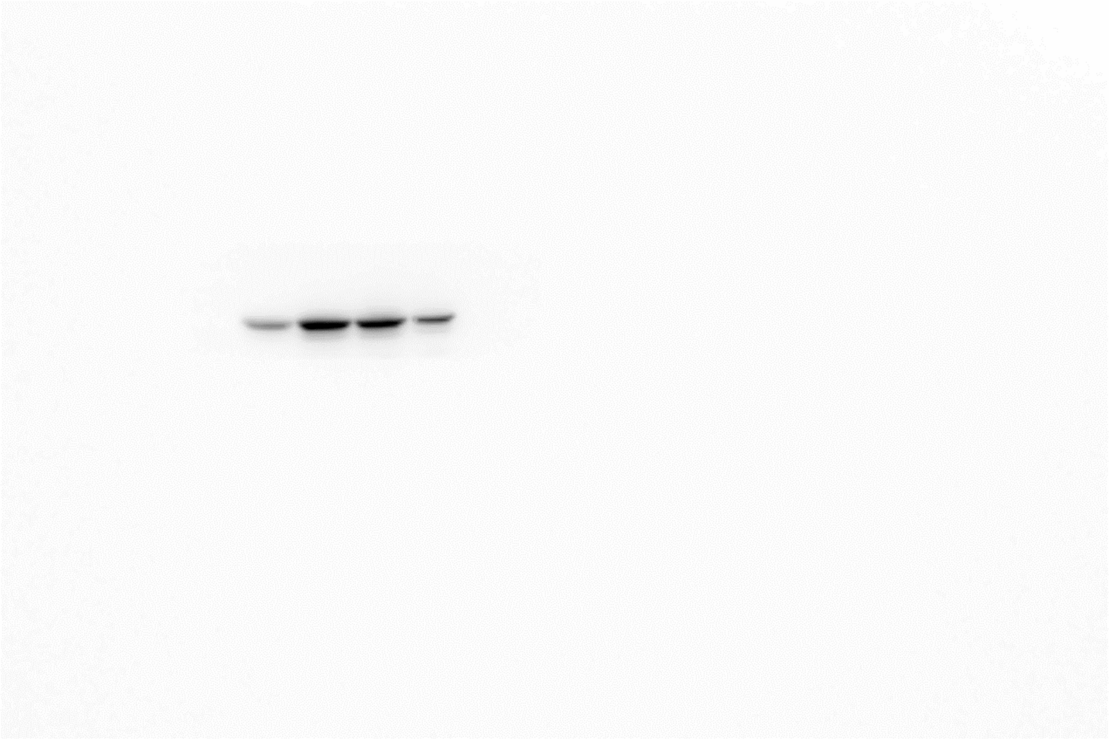

Gel photography of p-p65 protein in nuclear extracts:

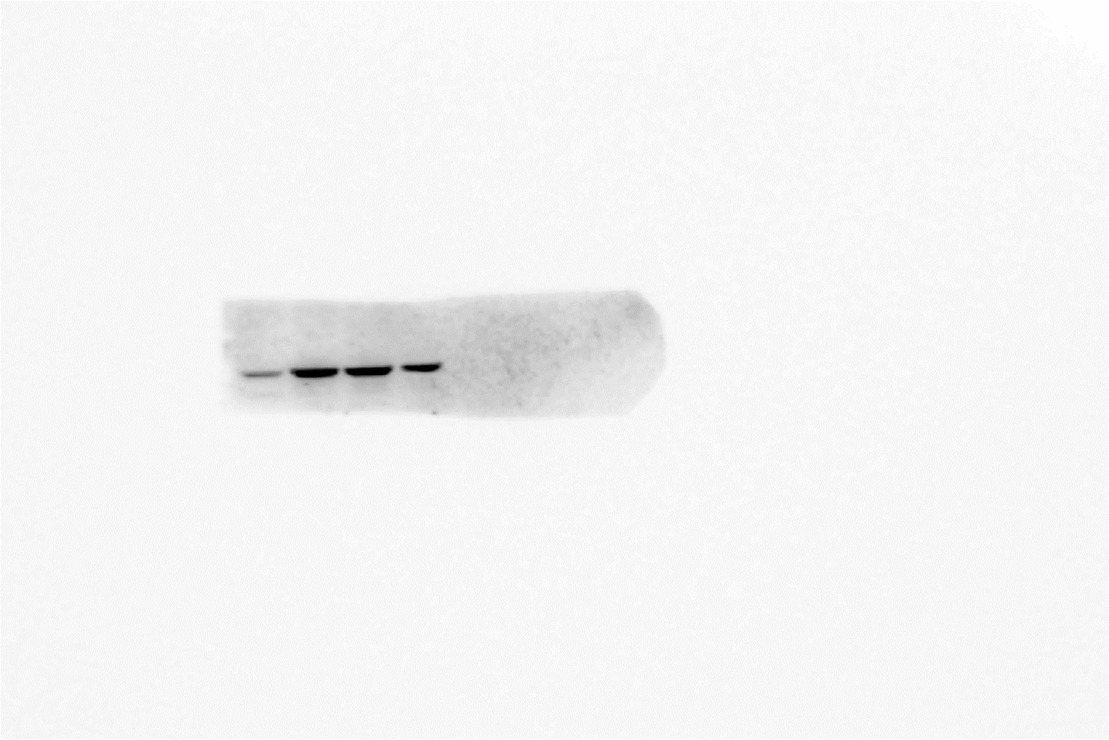

Gel photography of Histone H3 protein in nuclear extracts:

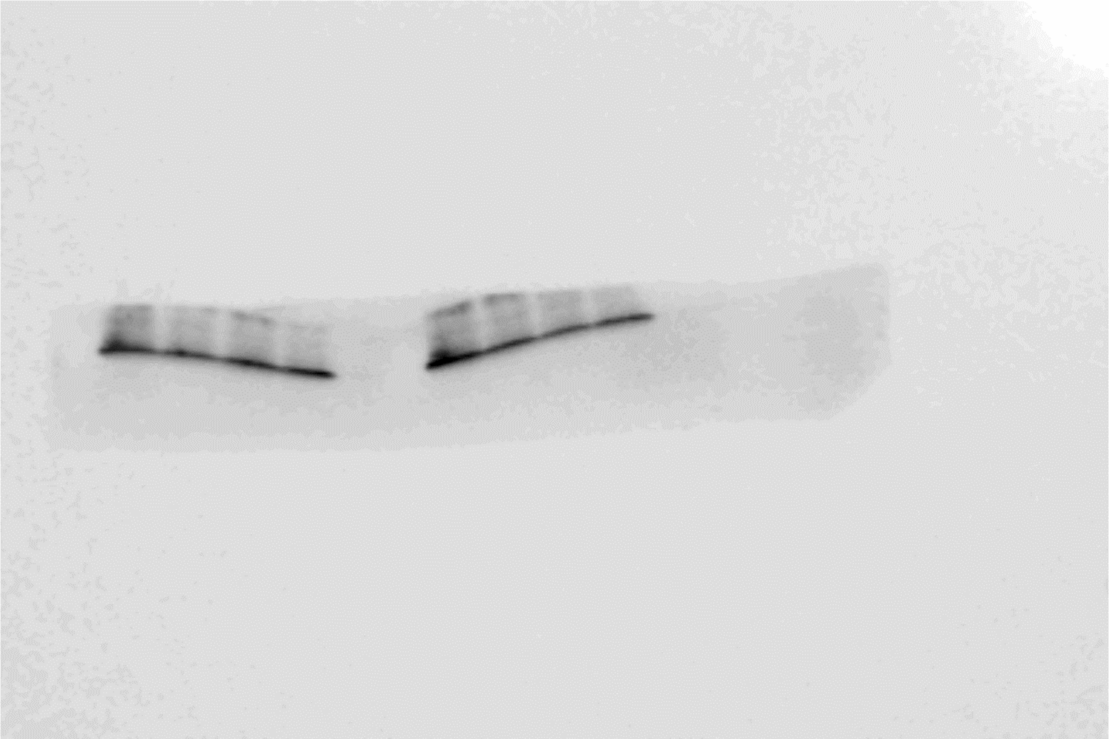

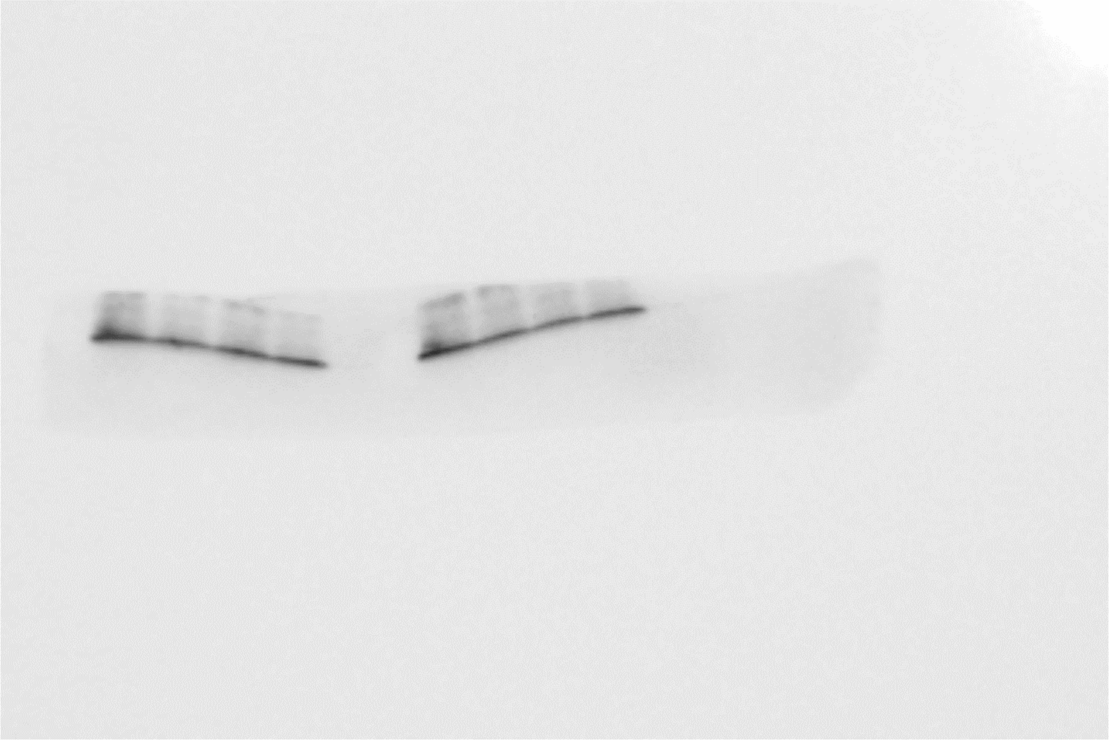

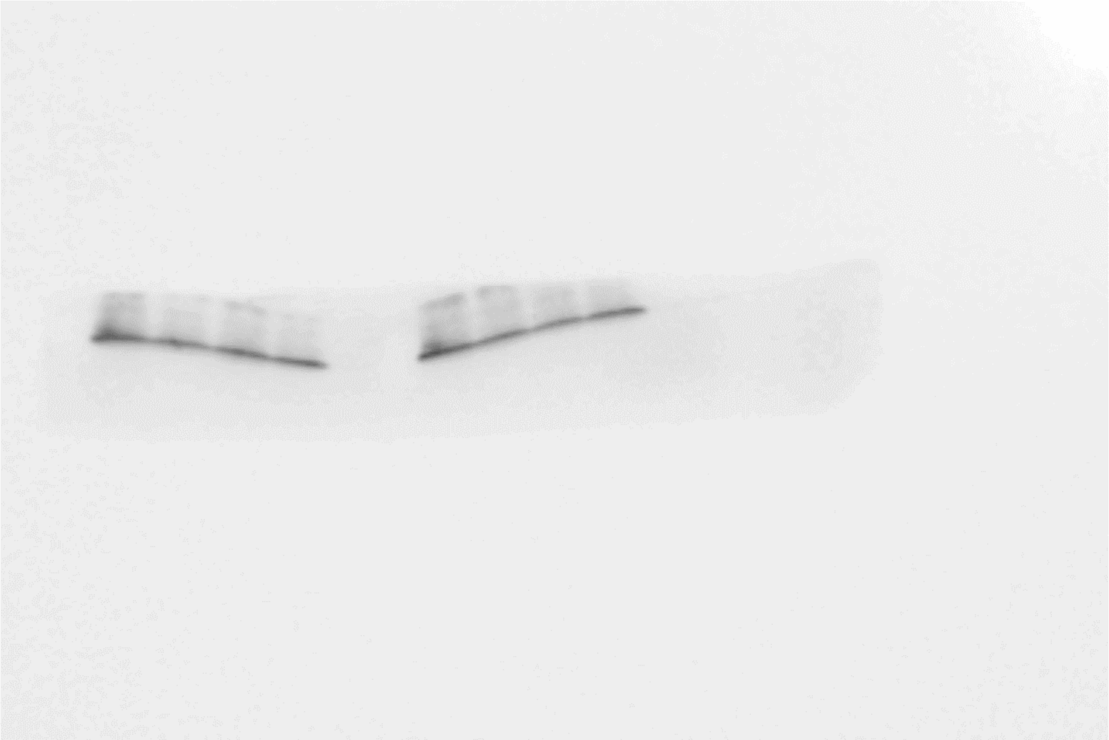

Gel photography of p65 protein in cytoplasmic extracts:

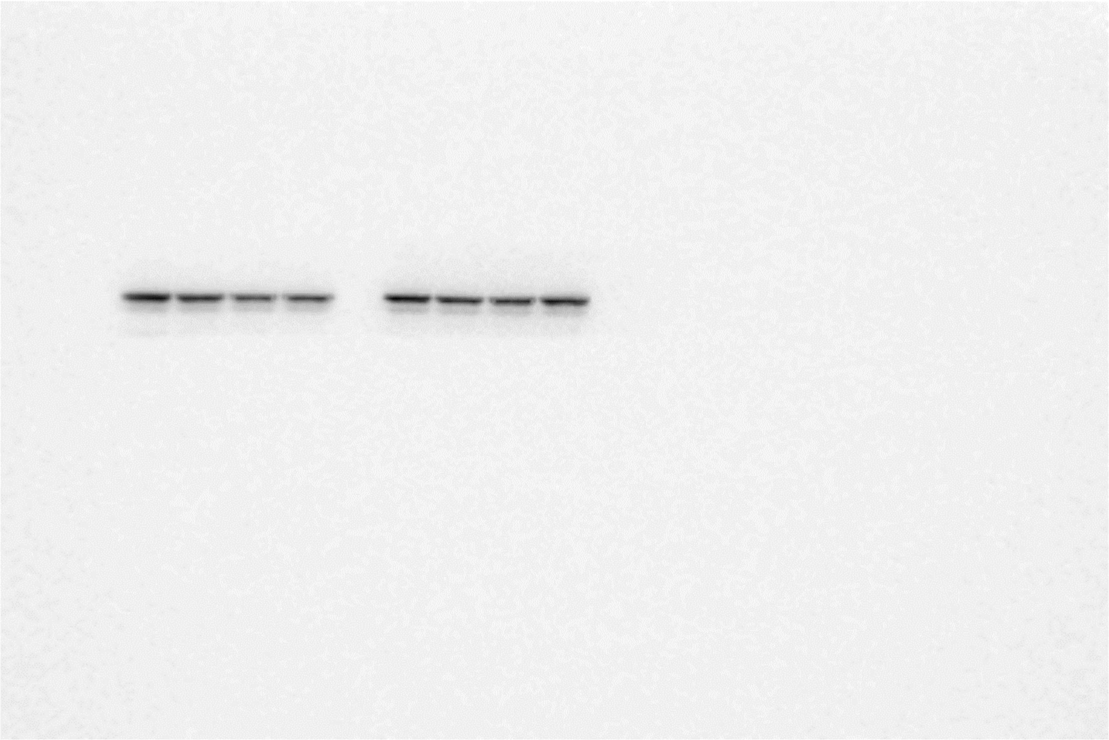

Gel photography of β-actin protein in cytoplasmic extracts:

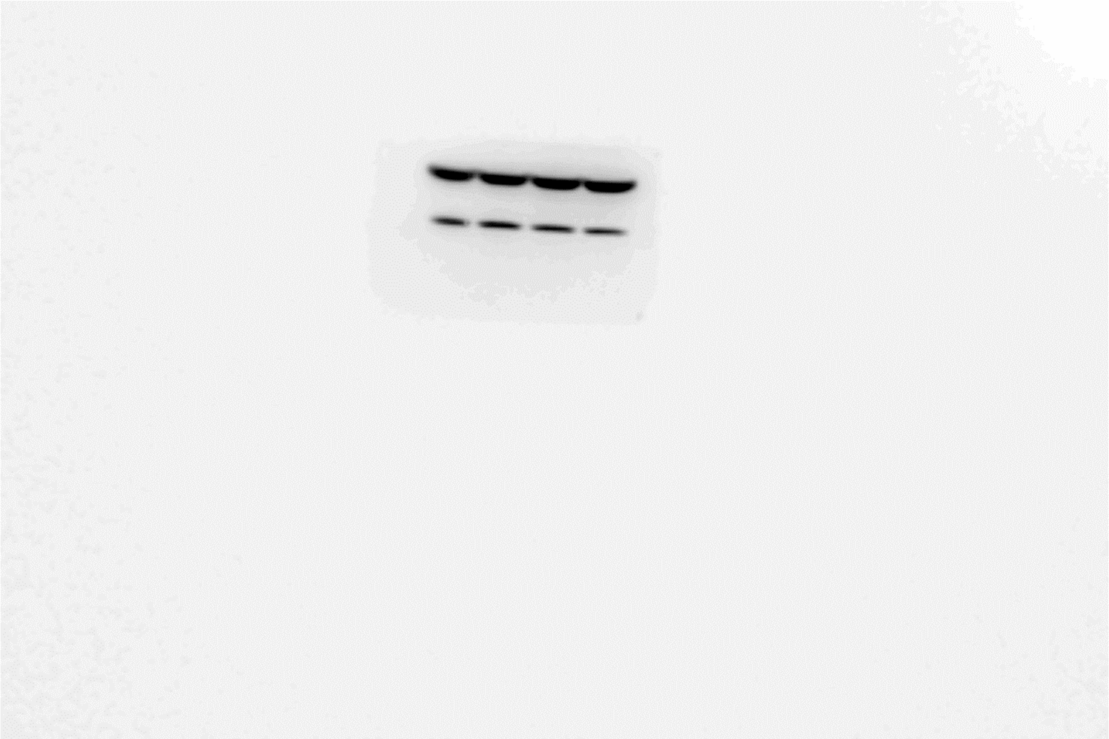

Supplement: Supplementary file 1 — Supplementary information [file 41598_2018_21783_MOESM1_ESM.pdf]
